# P-218. Treatment of Healthcare Facility-Onset *Clostridioides difficile* Infection Among Patients with Toxin Negative Stool Molecular Assay Results

**DOI:** 10.1093/ofid/ofae631.422

**Published:** 2025-01-29

**Authors:** Olivia Z Mobarakai, Polly van den Berg, Kara Reid, Adrienne Terico, Onyeka Nwankwo

**Affiliations:** Pennsylvania Hospital, Philadelphia, Pennsylvania; University of Pennsylvania, Philadelphia, Pennsylvania; Pennsylvania Hospital, Philadelphia, Pennsylvania; Pennsylvania Hospital, Philadelphia, Pennsylvania; Pennsylvania Hospital, Philadelphia, Pennsylvania

## Abstract

**Background:**

CDC's National Healthcare Safety Network (NHSN) defines a healthcare facility-onset *Clostridioides difficile (C. diff)* infection (HO-CDI) as a positive stool test result for *C. diff* toxin collected on or after hospital day (HD) 4. Currently, using two-stage algorithm testing, a nucleic acid amplification test (NAAT) that results positive for *C. diff* gene and negative for *C. diff* toxin (NAAT+/Toxin-) does not meet the HO-CDI definition as this may represent colonization. However, many NAAT+/Toxin- patients receive treatment given concern for clinical CDI. NHSN has proposed updating the healthcare facility-onset definition to include NAAT+/Toxin- patients who receive ≥ 5 days of antibiotic treatment for CDI within 2 calendar days (HT-CDI). To understand the increase in healthcare facility-onset CDI that may occur following this definition change, we describe the incidence of treated NAAT+/Toxin- patients at our hospital and examine factors associated with treatment.

**Figure:**
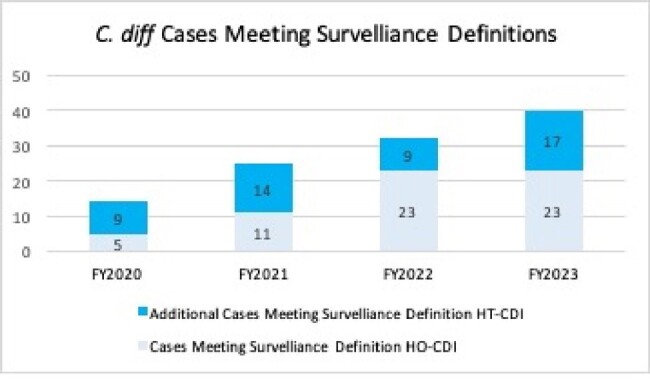

**Methods:**

Retrospective, observational cohort study evaluating patients ≥ 18 yo admitted between 7/2019 and 6/2023 with a NAAT+/Toxin- test collected on or after HD 4. Patients were identified using Theradoc software and variables were extracted by chart review. Comparisons between groups were performed using Fisher’s exact test.

**Figure:**
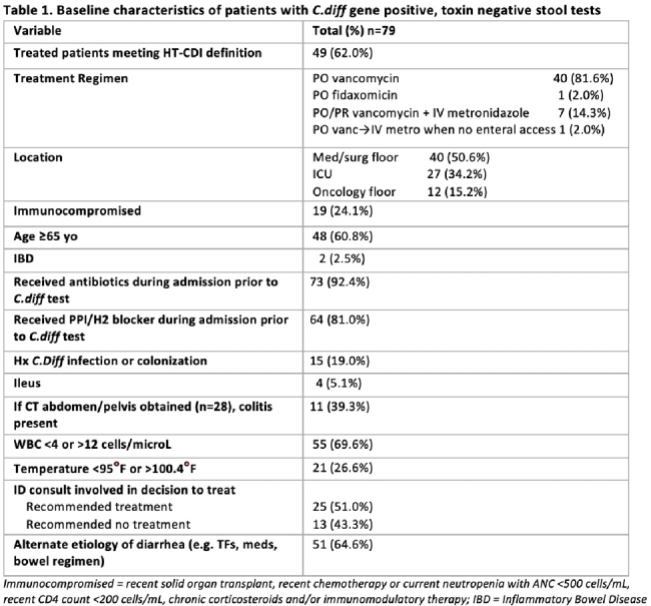

**Results:**

49 of 79 (62.0%) patients with NAAT+/Toxin- results received treatment for CDI and met the HT-CDI definition. There would have been 9, 14, 9, and 17 cases added during FY 2020, 2021, 2022, 2023 respectively (Figure 1). WBC was the only variable associated with treatment of NAAT+/Toxin- diarrhea (Table 1).

**Figure:**
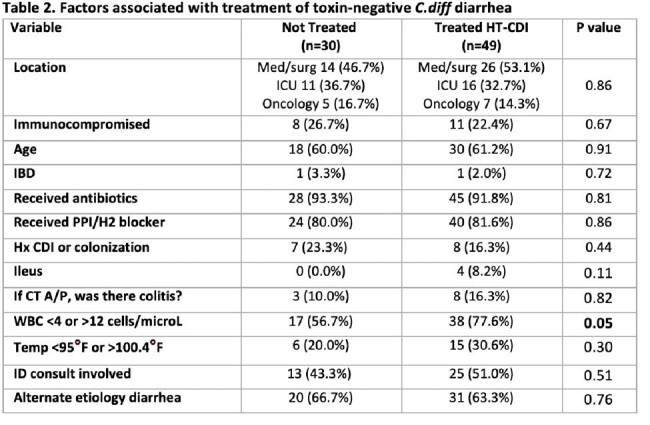

**Conclusion:**

62.0% of patients with *C.diff* NAAT+/Toxin- diarrhea were treated for suspected CDI. The majority (64.6%) of patients had a documented alternative etiology for diarrhea and often lacked associated symptoms of CDI (e.g., fever, colitis). While the purpose of NHSN’s creation of the HT-CDI definition was to improve upon the existing measure of HO-CDI, our data suggests that incorporating a provider’s decision to treat may not be the best way to discern if NAAT+/Toxin- patients have CDI. Our study highlights the importance of diagnostic stewardship and provider education on incorporating relevant signs and symptoms of CDI into decision making regarding treatment.

**Disclosures:**

**All Authors**: No reported disclosures

